# A summary of the Japan septic disseminated intravascular coagulation study

**DOI:** 10.1002/ams2.326

**Published:** 2018-01-10

**Authors:** Mineji Hayakawa, Kota Ono

**Affiliations:** ^1^ Emergency and Critical Care Center Hokkaido University Hospital Sapporo Japan; ^2^ Clinical Research and Medical Innovation Center Hokkaido University Hospital Sapporo Japan

**Keywords:** coagulopathy, sepsis/multiple organ failure

## Abstract

Over the past few decades, the large, international, randomized controlled trials of anticoagulant therapies for patients with sepsis have not yielded any improvement in mortality rates. However, in Japan, anticoagulant therapies are administered for sepsis patients with disseminated intravascular coagulation (DIC), but not for sepsis patients without DIC. Furthermore, epidemiological data regarding sepsis in Japan are scarce. Therefore, a nationwide multicenter retrospective observational study, the Japan Septic Disseminated Intravascular Coagulation (JSEPTIC DIC) study, was undertaken. The JSEPTIC DIC study enrolled 42 intensive care units and included 3,195 patients with sepsis. The results of the JSEPTIC DIC study indicated the following: (i) anticoagulant therapy may be effective in sepsis‐induced DIC patients at high risk for death, (ii) recombinant human soluble thrombomodulin administration and antithrombin supplementation are associated with survival benefits in patients with sepsis‐induced DIC.

## Introduction

In cases of sepsis and septic shock, the dysregulation of systemic coagulation and fibrinolytic systems frequently leads to disseminated intravascular coagulation (DIC).^1^, ^2^, ^3^ Moreover, DIC often induces multiple organ failure, which is associated with a high mortality rate, owing to the development of microthrombi that cause tissue hypoperfusion.^1^, ^2^, ^3^

Over the past few decades, although some anticoagulant agents, such as antithrombin (AT), tissue factor pathway inhibitor, and activated protein C, have been investigated for use in patients by international large randomized controlled trials (RCTs), no remarkable effect on the mortality rate has been reported.^4^, ^5^, ^6^, ^7^ However, the post‐hoc subgroup analyses of those RCTs indicated that the anticoagulant therapies resulted in improved mortality rates in patients with sepsis‐induced DIC.^8^, ^9^ In Japan, anticoagulant therapy has been approved for use as adjunctive therapy for patients with sepsis‐induced DIC and is now widely applied in clinical settings. However, this approach is not practiced in cases of sepsis without DIC. A recent meta‐analysis of RCTs reported that the survival benefit of anticoagulant therapy was observed only in sepsis patients with DIC, but not in those without DIC.^10^ Furthermore, although many epidemiological studies for sepsis have been carried out,^11^, ^12^, ^13^, ^14^, ^15^ epidemiological data regarding sepsis in Japan is limited.^16^, ^17^

Therefore, we undertook a nationwide multicenter retrospective observational study, named the Japan Septic Disseminated Intravascular Coagulation (JSEPTIC DIC) study, which reported the effects of anticoagulant therapy on sepsis in real world clinical settings.^18^, ^19^, ^20^, ^21^ Herein, we present a summary of the JSEPTIC DIC study.

## Basic information regarding the JSEPTIC DIC study

The JSEPTIC DIC study involved 42 intensive care units (ICUs) from 40 institutions throughout Japan. The ICU characteristics are presented in Table 1. The JSEPTIC DIC study included 3,195 consecutive adult patients with severe sepsis or septic shock, which was defined on the basis of the International Sepsis Definitions Conference criteria,^22^ diagnosed between January 2011 and December 2013. The JSEPTIC DIC study excluded patients who were <16 years old or patients who developed severe sepsis or septic shock following ICU admission.

**Table 1:** Characteristics of the intensive care units (ICUs) that participated in the Japan Septic Disseminated Intravascular Coagulation study

	*n* = 42
Hospital characteristics	
University hospital	22 (52.4)
Other	20 (47)
ICU characteristics	
General ICU	24 (57.1)
Emergency ICU	18 (42.9)
ICU policy	
Closed policy	17 (40.5)
Open policy	18 (42.9)
Other	7 (16.7)
Number of beds	11 (8–15)

Data are presented as median (interquartile range) or *n* (%).

## Characteristics, treatments, and outcomes of patients with sepsis treated in the ICU

The JSEPTIC DIC study included 1,916 men (60%) and 1,279 women (40%). The mean age was 70 ± 15 years. The mean Acute Physiology and Chronic Health Evaluation II score was 23 ± 9. The median Sequential Organ Failure Assessment (SOFA) score was 9 (interquartile range, 6–12). The primary infection sites are presented in Table 2. The in‐hospital mortality rate was 33%. These characteristics of the septic patients treated in the ICU were almost the same as that previously reported in Japanese published works.^16^, ^17^

**Table 2:** Primary infection site responsible for the sepsis as reported by intensive care units participating in the Japan Septic Disseminated Intravascular Coagulation study

	Total *n* = 3,195
Abdomen	1,032 (32)
Lung/thorax	827 (26)
Urinary tract	509 (16)
Bone/soft tissue	374 (12)
Cardiovascular system	68 (2)
Central nervous system	63 (2)
Catheter‐related	44 (1)
Other	60 (2)
Unknown	218 (7)

Data are expressed as *n* (%).

## Optimal targets of anticoagulant therapy in sepsis

**Figure 1:**
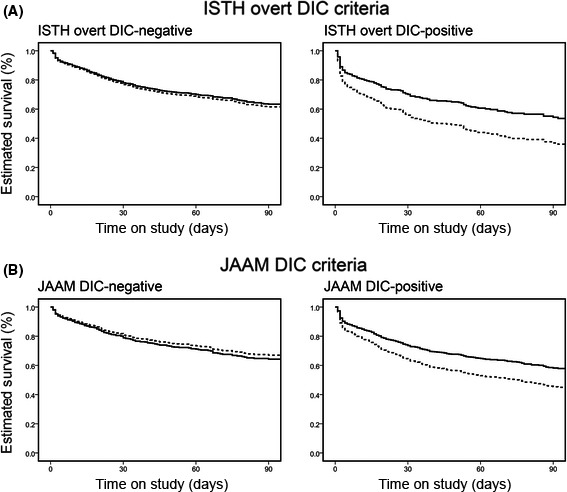
Adjusted estimated survival curves in patients with or without disseminated intravascular coagulation (DIC). A, DIC was diagnosed on the basis of the International Society on Thrombosis and Haemostasis (ISTH) criteria for overt DIC. Significant associations between anticoagulant therapy and lower in‐hospital mortality rates were observed only in patients with DIC (adjusted hazard ratio [HR], 0.609; 95% confidence interval [CI], 0.456–0.814; *P* = 0.001), whereas mortality rates in patients without DIC were not different regardless of anticoagulant therapy (adjusted HR, 0.941; 95% CI, 0.773–1.145; *P* = 0.543). B, DIC was diagnosed on the basis of the Japanese Association for Acute Medicine (JAAM) DIC criteria. Significant associations between anticoagulant therapy and lower in‐hospital mortality rates were observed only in patients with DIC (adjusted HR, 0.685; 95% CI, 0.559–0.839; *P* < 0.001), whereas mortality rates in patients without DIC were not different regardless of anticoagulant therapy (adjusted HR, 1.104; 95% CI, 0.839–1.453; *P* = 0.478). Dotted line, patients in the control group; solid line, patients in the anticoagulant group. Cited as Figure 3 in our previous report.^19^

**Figure 2:**
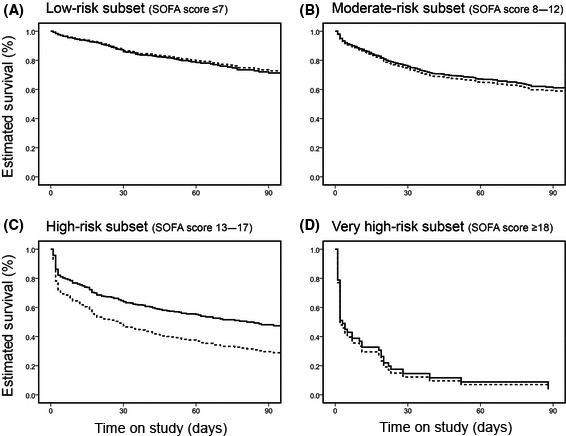
Adjusted estimated survival curves in four subsets of patients with sepsis‐induced disseminated intravascular coagulation, stratified according to baseline Sequential Organ Failure Assessment (SOFA) score. A, Low‐risk subset (SOFA score ≤7): adjusted hazard ratio [HR], 1.063; 95% confidence interval [CI], 0.716–1.580; *P* = 0.761. B, Moderate‐risk subset (SOFA score 8–12): adjusted HR, 0.927; 95% CI, 0.728–1.181; *P* = 0.540. C, High‐risk subset (SOFA score 13–17): adjusted HR 0.601; 95% CI, 0.451–0.800; *P* < 0.001. D, Very high‐risk subset (SOFA ≥18): adjusted HR 0.915; 95% CI, 0.418–2.003; *P* = 0.825. However, this analysis was not definitive because of the small sample sizes of the subsets. Dotted line, patients in the control group; solid line, patients in the anticoagulant group. Cited as Figure 4 in our previous report.^19^

We previously reported that anticoagulant therapy is significantly associated with lower in‐hospital mortality in sepsis patients with DIC, but not in sepsis patients without DIC (Fig. 1).^19^ We found that the associations between anticoagulant therapy and lower in‐hospital mortality were not significant, regardless of the DIC diagnosis criteria applied. We also evaluated the relationship between the effects of anticoagulant therapy and disease severity. A significant association between anticoagulant therapy and lower in‐hospital mortality was observed in high‐risk sepsis patients (SOFA score 13–17) but not in low‐risk to moderate‐risk sepsis patients (SOFA score ≤12; Fig. 2). These results indicate that anticoagulant therapies may be effective in sepsis‐induced DIC patients at high risk of death.

## Recombinant human soluble thrombomodulin

Recombinant human soluble thrombomodulin (rhTM), like AT, is frequently used as an anticoagulant for treating DIC.^18^, ^23^ Thrombomodulin is a receptor of thrombin and protein C on the endothelial cell surface and plays an important role in the regulation of coagulation and the innate immune system.^24^ Recombinant human soluble thrombomodulin was developed and approved in Japan in 2008 for treating patients with DIC.^25^ However, there is very limited clinical evidence supporting the use of rhTM in patients with sepsis‐induced DIC. Therefore, we evaluated the effect of rhTM treatment on sepsis‐induced DIC using a propensity score analysis for the dataset of the JSEPTIC DIC study.^20^

**Figure 3:**
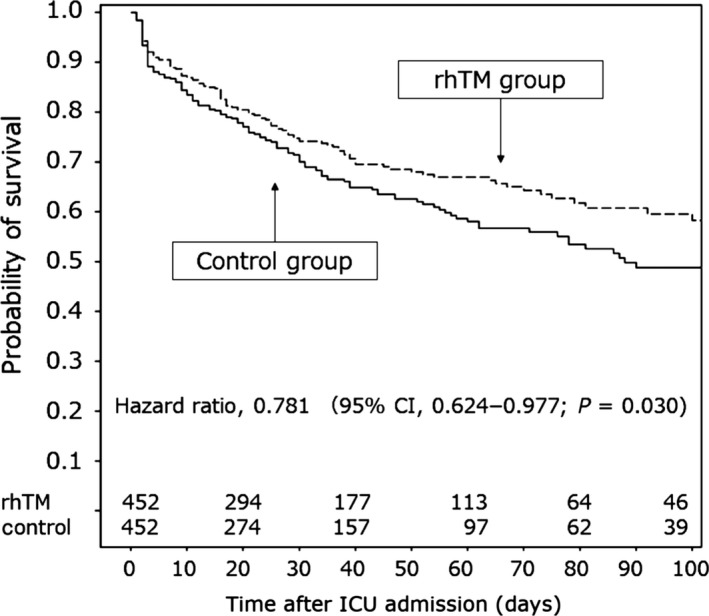
Survival time curves for patients with sepsis‐induced disseminated intravascular coagulation in propensity score‐matched groups according to treatment with recombinant human soluble thrombomodulin (rhTM group) or without (control group). The survival rate was higher in the rhTM group than in the control group. Cited as Figure 3 in our previous report.^20^

We selected 1,784 patients with sepsis‐induced DIC from the 3,195 patients in the JSEPTIC DIC study. Among these patients, 645 were given rhTM (rhTM group) and 1,139 patients were not (control group). Propensity score matching yielded 452 matched pairs, after which the characteristics and therapeutic interventions of the two groups were appropriately balanced. Lower in‐hospital all‐cause mortality was significantly associated with rhTM administration according to the three propensity score analyses (propensity score matching, inverse probability of treatment weighting, and quintile‐stratified analyses). Survival time analysis revealed a higher survival rate in the propensity score‐matched rhTM group than in the propensity score‐matched control group (hazard ratio, 0.781; 95% confidence interval, 0.624–0.977; *P* = 0.03) (Fig. 3).^20^ These results revealed that rhTM administration was associated with reduced in‐hospital mortality among patients with sepsis‐induced DIC.

## Antithrombin

Antithrombin is among the most important physiologic anticoagulants.^26^ However, in sepsis‐induced DIC, a decrease in AT activity is frequently observed^27^, ^28^, ^29^, ^30^ and is associated with high mortality rates.^31^, ^32^ Several RCTs to investigate the effects of high‐dose AT administration in patients with sepsis have been carried out.^4^, ^33^, ^34^, ^35^, ^36^ Although some RCTs indicated benefits of high‐dose AT administration in patients with sepsis,^33^, ^34^, ^35^ a large RCT (the KyberSept trial) failed to show any survival benefits.^4^ However, a subgroup analysis of the KyberSept trial indicated that AT administration significantly improved survival rates in patients with sepsis‐induced DIC.^9^

In Japan, a supplemental dose of AT (4,500 IU over 3 days) is widely given to patients with sepsis‐induced DIC and low AT levels in clinical settings, although high‐dose AT is not recommended as treatment for patients with sepsis‐induced DIC. Recently, two studies that used a nationwide administrative database reported that AT supplementation was beneficial in treating sepsis‐induced DIC.^37^, ^38^ However, the evidence for this practice is insufficient. Therefore, we analyzed the effect of AT supplementation in patients with sepsis‐induced DIC using propensity score analyses based on the JSEPTIC DIC study dataset.^21^

We selected 1,784 patients with sepsis‐induced DIC from among 3,195 patients in the JSEPTIC DIC study, similar to the approach used for the analysis of rhTM effects.^20^ Among these patients, 715 were given rhTM (rhTM group) and 1,069 patients were not (control group). Propensity score matching created 461 matched pairs, and the characteristics and therapeutic interventions of the two groups were appropriately balanced. The three propensity score analyses (propensity score matching, inverse probability of treatment weighting, and quintile‐stratified analyses) revealed the same tendency: lower in‐hospital all‐cause mortality was associated with AT administration. However, statistically significant differences were not clearly observed. The survival rates in the matched groups were not different.^21^

**Figure 4:**
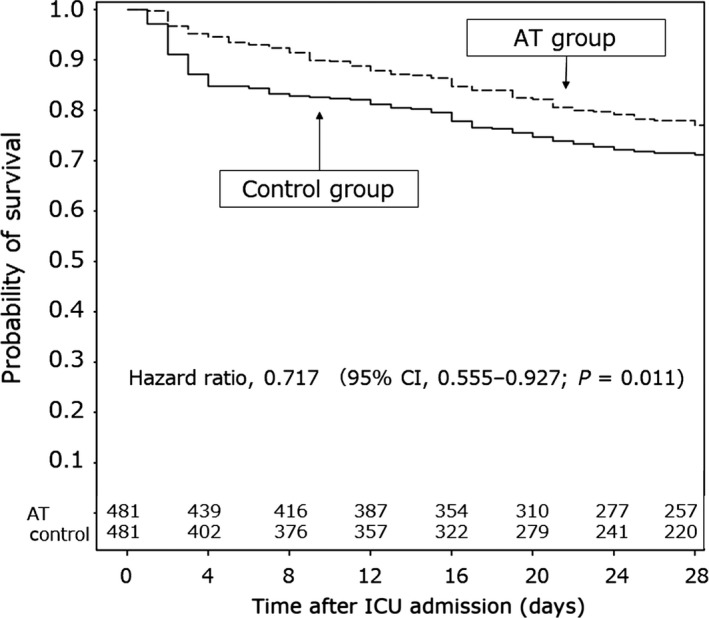
Survival time curves over the 28‐day period after admission to the intensive care unit (ICU) for patients with sepsis‐induced disseminated intravascular coagulation. Patients were allocated to groups according to treatment with antithrombin (AT group) or without (control group) in the re‐analysis. In the original analysis,^21^ the observation period was the duration of hospital stay (not restricted). In the re‐analysis, the observation period was restricted to a 28‐day period after ICU admission. Survival time analysis revealed that the survival rate was higher in the propensity score‐matched AT group than in the propensity score‐matched control group (hazard ratio, 0.717; 95% confidence interval, 0.555–0.927, *P* = 0.011).

Some experts indicated that an analysis of the survival benefits of AT supplementation therapy over a short period, such as 28 days after ICU admission, yielded more concrete results than that conducted over a long period (the results reported in our previous study^21^). Therefore, we re‐analyzed the survival benefits of AT supplementation therapy over the 28‐day period after ICU admission. Survival time analysis revealed higher survival rates in the propensity score‐matched AT group than in the propensity score‐matched control group (hazard ratio, 0.717; 95% confidence interval, 0.555–0.927; *P* = 0.011) (Fig. 4). These results revealed that AT supplementation was associated with reduced in‐hospital mortality among patients with sepsis‐induced DIC.

## Conclusions

The results of the JSEPTIC DIC study indicated that anticoagulant therapies, mainly rhTM and AT, were associated with survival benefits among patients with sepsis‐induced DIC in real world clinical settings.

## Disclosure

Mineji Hayakawa received a grant for the basic research and a lecturer's fee from Asahi Kasei Pharma Co. The other authors have no conflict of interest.
